# Metagenomic Analysis for Evaluating Change in Bacterial Diversity in TPH-Contaminated Soil after Soil Remediation

**DOI:** 10.3390/toxics9120319

**Published:** 2021-11-24

**Authors:** Jin-Wook Kim, Young-Kyu Hong, Hyuck-Soo Kim, Eun-Ji Oh, Yong-Ha Park, Sung-Chul Kim

**Affiliations:** 1 Department of Bio-Environmental Chemistry, Chungnam National University, Daejeon 34134, Korea; kin1888@naver.com (J.-W.K.); hyk102030@naver.com (Y.-K.H.); 2 Department of Biological Environment, Kangwon National University, Chuncheon 24341, Korea; khs25@kangwon.ac.kr; 3 Korea Environment Institute, Sejong 30147, Korea; ejoh@kei.re.kr

**Keywords:** TPH, soil remediation, illumina sequencing, bacterial diversity, bacterial community

## Abstract

Soil washing and landfarming processes are widely used to remediate total petroleum hydrocarbon (TPH)-contaminated soil, but the impact of these processes on soil bacteria is not well understood. Four different states of soil (uncontaminated soil (control), TPH-contaminated soil (CS), after soil washing (SW), and landfarming (LF)) were collected from a soil remediation facility to investigate the impact of TPH and soil remediation processes on soil bacterial populations by metagenomic analysis. Results showed that TPH contamination reduced the operational taxonomic unit (OTU) number and alpha diversity of soil bacteria. Compared to SW and LF remediation techniques, LF increased more bacterial richness and diversity than SW, indicating that LF is a more effective technique for TPH remediation in terms of microbial recovery. Among different bacterial species, Proteobacteria were the most abundant in all soil groups followed by Actinobacteria, Acidobacteria, and Firmicutes. For each soil group, the distribution pattern of the Proteobacteria class was different. The most abundant classed were Alphaproteobacteria (16.56%) in uncontaminated soils, Deltaproteobacteria (34%) in TPH-contaminated soils, Betaproteobacteria (24%) in soil washing, and Gammaproteobacteria (24%) in landfarming, respectively. TPH-degrading bacteria were detected from soil washing (23%) and TPH-contaminated soils (21%) and decreased to 12% in landfarming soil. These results suggest that soil pollution can change the diversity of microbial groups and different remediation techniques have varied effective ranges for recovering bacterial communities and diversity. In conclusion, the landfarming process of TPH remediation is more advantageous than soil washing from the perspective of bacterial ecology.

## 1. Introduction

Soil pollution attributed to total petroleum hydrocarbons (TPHs) is a worldwide concern [1]. Organic pollutants such as gasoline and diesel can reach deep into the soil and aquifers resulting in soil and groundwater pollution and even causing economic losses and destruction of agricultural production systems [2,3]. Since the Soil Environment Conservation Act was implemented in 1995, the Korean government has promoted soil and groundwater remediation through monitoring of contaminated regions and applying remediation techniques [4].

Various physicochemical and biological techniques, such as stabilization, soil washing, electrokinetic remediation, landfarming, phytoremediation, and biodegradation are used to remediate TPH-contaminated soil [5]. Soil washing and landfarming, in particular, are widely used in the TPH soil remediation process, with numerous advantages including simplicity and cost-effectiveness [6,7,8,9]. Since the 1980s, soil washing has been extensively researched and applied throughout Europe and North America. This method is a physicochemical process that uses desorption and solubilization to remove contaminants sorbed to the soil and transfers them to the liquid phase [10]. Furthermore, landfarming is a biological remediation method that promotes aerobic biodegradation of soil bacteria to degrade petroleum hydrocarbons. Supplementing nutrients and water, cultivating the soil, and sometimes adding a bacterial consortia to improve the biodegradability of contaminants are all part of this process [11,12].

The capacity for soil washing and landfarming processes to remove TPH efficiently has been well documented. There are, however, few studies on the biological properties of soil such as soil bacterial diversity and changes in the community structure after the soil remediation process. Recent study showed that chemical oxidation technique for TPH-contaminated soil had an adverse effect on diversity and activity of microbes [13]. However, most of study was conducted in lab-scale experiment and lack of information is available for change of indigenous soil microbial activity with field experiments.

Soil bacteria do not live in isolation; they coexist with many other species and interact organically with the soil environment. They also play an important role in soil quality improvement, such as circulating nutrients and decomposing organic material [14,15,16]. Soil bacteria are an essential component of the soil ecosystem because of these features and it is critical to understand not just the chemical properties of the soil, but also biological properties such as the soil microbial cluster structure.

According to current knowledge, approximately 0.3% of soil bacteria can be cultured in media, and the majority of the remaining bacteria are thought to be difficult or impossible to cultivate using standard procedures [17]. The culture-dependent method cannot accurately analyze the structure of bacterial communities in the soil because of this constraint. However, with the development of molecular biology techniques, culture-independent methods such as denaturing gradient gel electrophoresis (DGGE), restriction fragment length polymorphism (RFLP), and fluorescence in situ hybridization (FISH) based on bacterial 16S rRNA gene sequences, have been developed [18,19,20]. They have been widely utilized to identify bacterial communities instead of culture-dependent methods Furthermore, with the recent emergence and development of next-generation sequencing (NGS) technologies, research on metagenomic analysis to identify the overall structure of the bacterial community is being actively conducted in various fields [21,22,23].

The aim of this study was to use metagenomic analysis to determine how the bacterial community changed in response to TPH contamination and soil remediation processes. Four types of soil were obtained from the soil remediation facility, and the Illumina MiSeq platform was used to analyze bacterial diversity and communities.

## 2. Materials and Methods

### 2.1. Soil Sampling and Sample Description

Soil samples were collected from a closed military base located in Gyeonggi Province, South Korea. The petroleum carbonate threshold value (2000 mg/kg) set by the Korea Ministry of Environment (KME) was exceeded in this area, and two soil remediation processes were used to clean up the polluted soil: soil washing and landfarming. A total of 11 soil samples were collected from the site including: two control samples, four from petroleum-contaminated sites (CS), three samples obtained after the soil washing process (SW), and two from the landfarming process (LF). Three sub-soil samples were taken at a depth of 0–20 cm from each sampling site and combined to form one sample. Each sample was thoroughly homogenized by manual shaking. The soils were sieved and separated into three subsamples using a 2 mm mesh. The first soil sample was air-dried at 35 °C for physicochemical analyses. The remaining subsoil sample was stored at 4 °C for petroleum carbonate analysis. Another ten grams of fresh soil was transferred to a 50 mL conical tube and stored at −80 °C for DNA extraction.

### 2.2. Soil Chemical Properties and Total Petroleum Hydrocarbon (TPH) Analysis

Soil pH and electrical conductivity (EC) were measured in a soil/distilled water ratio of 1:5 (*w*/*v*) using a pH meter (Orion Star™ A111, Thermo Fisher Scientific, Waltham, MA, USA) and an EC meter (SevenCompact Conductivity Meter S230, Mettler Toledo, Columbus, OH, USA) according to the Korean Standard Test Method (KSTM ES 07302). Soil organic matter (SOM) was analyzed using the Walkley–Black method [24]. Available phosphorus was extracted using Bray No. 1 [25], determined by the molybdenum blue method [26], and quantified using a UV-spectrophotometer (Uvmini-1240, Shimadzu, Kyoto, Japan). The cation exchange capacity (CEC) was extracted using the ammonium acetate method (pH 7.0) and determined by ICP-OES (Optima 3200XL, Perkin Elmer, Waltham, MA, USA). Total nitrogen was measured using a Kjeldahl distillation apparatus. Total petroleum hydrocarbon (TPH) in the soil samples was analyzed according to KSTM ES 07552. Briefly, 10 g of soil sample was extracted with 200 mL of dichloromethane and the extract was analyzed using a gas chromatograph flame ionization detector (7890A, Agilent, Santa Clara, CA, USA).

### 2.3. Metagenomic Analysis

#### 2.3.1. DNA Extraction, Polymerase Chain Reaction (PCR) Amplification, and 16S rRNA Gene Amplicon Sequencing

Genomic DNA from soil bacteria was extracted using the Nuclear Spin^®^ Soil DNA extraction kit, following the manufacturer’s guidelines (Macherey-Nagel, Düren, Germany). Briefly, 0.3 g of the soil sample was placed in MN Bead Tubes Type A containing ceramic beads, 700 μL of lysis buffer SL1 and 150 μL of enhancer SX were added, and bead-beating was performed using a Mini Beadbeater-16 (Macherey-Nagel, Düren, Germany). The subsequent process was performed according to the manufacturer’s protocol, and the genomic DNA was extracted with 50 μL of Elution Buffer SE. The concentrations and purities of the extracted DNA were evaluated using a UV-Vis spectrophotometer (Optizen NANO Q, Mecasys, Daejeon, Korea) and stored at −80 °C until further analysis. Genomic DNA samples were sent to LabGenomics (Seongnam, Korea) for metagenomic analysis, and PCR amplification and Illumina MiSeq sequencing were performed. To analyze the soil bacterial colony structure, the universal primers 341F (5’-CCTACGGNGGCWGCAG-3’) and 805R (5’-GACTACHVGGGGTATCC-3’) were used to amplify the V3–V4 regions of the 16S rRNA. PCR was performed using Herculase II Fusion DNA Polymerase (Agilent, Santa Clara, CA, USA), and carried out in a total volume of 23 μL containing: 2.5 μL amplicon PCR F, R primer, 0.5 μL Herculase ΙΙ Fusion DNA Polymerase, 5.0 μL 5 × Herculase ΙΙ reaction buffer, 0.25 μL dNTPs (100 nM), and 14.75 μL PCR-grade water. Amplicon PCR conditions were as follows: an initial denaturation step of 3 min at 95 °C, followed by 25 cycles of 95 °C for 30 s, 55 °C for 30 s, 72 °C for 30 s, and a final extension for 5 min at 72 °C. Then, limited-cycle amplification was performed to add multiplexing indices and Illumina adapter sequences, and the conditions were as follows: initial denaturation step for 3 min at 95 °C followed by eight cycles of 95 °C for 30 s, 55 °C for 30 s, 72 °C for 30 s, and a final extension for 5 min at 72 °C. The final product was normalized and pooled using PicoGreen (Promega, Madison, WI, USA), and the size of the libraries was verified using the TapeStation DNA screentape D1000 (Agilent). Paired-end sequencing was performed using the MiSeq™ platform (Illumina, San Diego, CA, USA).

#### 2.3.2. Bioinformatic Analysis

The raw sequence data obtained by MiSeq sequencing analysis were classified by sample using the index sequence, and a FASTQ file was generated. Then, the paired-end data, separated by each sample, were assembled into a single sequence using FLASH (ver. 1.2.11) [27], and sequences with a length of less than 400 bp or more than 500 bp were removed. To exclude low-quality, ambiguous, chimeric sequences, which are deemed sequencing errors, the acquired sequences were processed using the CD-HIT-OTU program [27], an operational taxonomic unit (OTU) analysis program, based on CD-HIT-EST. Sequences were clustered into species-level OTUs with 97% sequence similarity. Taxonomic assignment was performed by comparing the representative sequence from each OTU to the reference database (NCBI 16S Microbial) using BLAST+ (ver. 2.9.0) [28]. Various bioinformatics analyses were performed using QIIME (ver. 1.9) with the obtained OTU findings and taxonomy information [29]. In addition, beta diversity between soil groups was obtained based on weighted UniFrac distance [30], principal coordinate analysis (PCoA), and unweighted pair group method with arithmetic mean (UPGMA) clustering was conducted to compare and visualize the diversity of bacterial communities in the four soil groups.

### 2.4. Data Analysis

Statistical tests were performed using the Statistical Package for Social Science (SPSS) version 26.0 (2021, SPSS Inc., Chicago, IL, USA). Soil physicochemical properties, TPH concentration, and bacterial relative abundance were measured in triplicate and expressed as mean and standard deviation. Analysis of variance (ANOVA) was performed to verify the statistical difference with the results to confirm the normality and homogeneity of the variance, followed by the post-hoc Duncan’s test (*p* < 0.05) for multiple comparisons between soil samples.

## 3. Results and Discussion

### 3.1. Soil Chemical Properties and TPH Concentration

The chemical properties and TPH concentrations of the soil samples are listed in Table 1. The soil pH was slightly acidic in the control (6.23) and contaminated sites (CS) (6.22), but after the remediation process, it increased to neutral for the landfarming (LF) (6.90) and soil washing processes (SW) (7.23). The increased soil pH in the LF and SW soils was attributed to the remediation process. The soil pH was adjusted to neutral with microbial feeding solution to optimize the microbial activity in the LF treatment. In addition, the surfactant’s alkaline property was used to detach TPH from the soil, which may have resulted in an increase in soil pH in the SW treatment.

When compared to the control, the SW treatment had significantly lower concentrations of soil organic matter (SOM) (0.81%), average phosphorus (Av. P) (2.86 mg/kg) and total nitrogen (TN) (0.13%). Soils with particle sizes less than 0.075 mm, mainly clay portion, were washed out during the SW process and consequently, SOM and Av. P sorbed on surface of clay particle were also removed causing a lower concentration of SOM and Av. P compared to control. In addition, in SW treatment, soil washing solutions, water, and surfactants reduced TN concentration.

The concentrations of TPH in the control and CS groups were 77 and 2690 mg/kg, respectively. In Korea, the TPH contamination criterion was set at 2000 mg/kg, and we confirmed that the TPH concentration in CS soil was higher than the threshold value TPH concentrations in SW and LF were significantly decreased to 111 and 249 mg/kg, after the soil remediation process, indicating removal effectiveness of 95.8% and 90.7%, respectively.

### 3.2. Soil Bacterial Diversity Analysis

A summary of soil bacterial diversity analysis, including the number of OTUs, Good’s coverage, Chao1, and Shannon index is shown in Table 2. A total of 985,196 raw read counts were obtained from 11 soil samples through MiSeq sequencing analysis of soil bacterial communities. Low-quality and chimeric sequences were removed through preprocessing and clustering using CD-HIT-OTU, resulting in between 13,360 and 44,406 reads. In addition, the filtered bacterial sequences were clustered into OTUs with a similarity level of 97%. As a result, OTUs were observed in the range of 263–1483 in all soil samples. The control group had the largest average number of OTUs (1265), while the TPH-affected CS group showed a significant decrease in OTUs with the lowest average number of 296. In SW and LF, an average of 305 and 815 OTUs were measured, respectively.

Good’s coverage estimator ranged from 97.90% to 99.99%, exceeding 97% in all soil samples, indicating that the sequencing depth was sufficient to identify the entire bacterial population in the soil [31]. Alpha diversity was calculated to analyze the bacterial species richness (Chao 1 index) and diversity (Shannon index) within soil samples under four different conditions., The Chao1 index (316) of SW was similar to that of CS, and the Shannon index (5.94) was slightly increased (*p* < 0.05). LF on the other hand, had significantly increased Chao1 (1000) and Shannon index (7.06) than CS (*p* < 0.05).

These results reveal that TPH leaching into the soil reduces the richness and diversity of bacteria in the soil. TPH contamination has also been shown to reduce the alpha diversity of soil bacteria in several studies [32,33]. We also confirmed that Chao1 and Shannon indices decreased significantly in TPH-contaminated soil (CS) compared to the control in this study. We also found that the alpha diversity of bacterial communities was affected differently by each soil remediation approach. When the LF process was used, alpha diversity indices in the soil were restored to control levels but no significant changes in biodiversity were seen when the SW process was applied to TPH-contaminated soil.

Soil biodiversity may be affected by different remediation processes. SW, for example, is a physicochemical method of remediation that employs washing solutions. Although washing solutions efficiently remove TPH from the soil, they have been shown to have a deleterious impact on soil bacterial activity and diversity by altering the soil’s physicochemical properties [34,35]. In contrast, the concentration of TPH in the soil decreases in the LF process due to volatilization or biodegradation [36,37]. In this procedure, organic matter and bacterial consortia are used for successful biodegradation and increased soil bacterial activity [38,39]. Previous research has also found that employing the LF process to reduce TPH restored bacterial diversity to pre-contamination levels [40].

**Figure 1:**
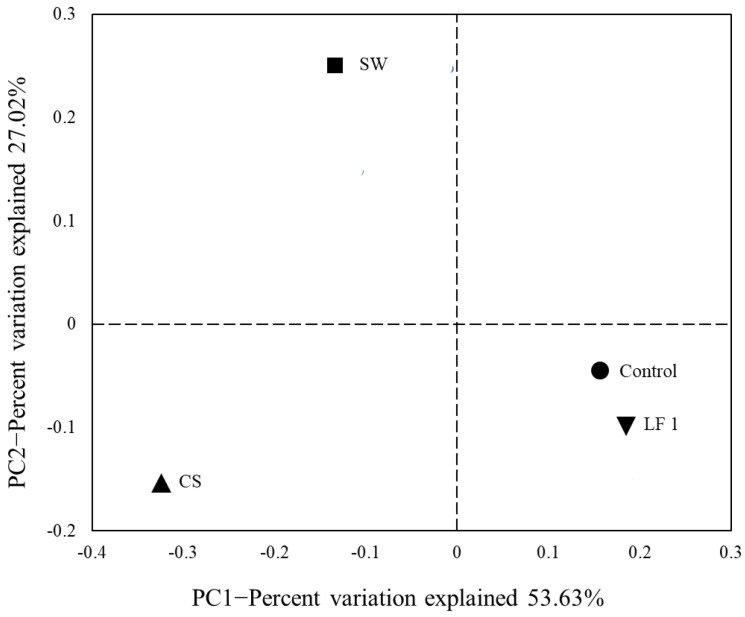
Principal coordinate analysis (PCoA) of bacterial communities in each soil samples.

The similarity of the four soil groups was compared using a beta diversity analysis. The distribution of bacterial communities in the four groups of soil samples was visually represented through PCoA (Figure 1). The first (53.63%) and second principal coordinates (27.02%) accounted for 80.65% of the total variation. The control and LF groups were clustered at a similar level, indicating that the bacterial communities were similar between the control and LF groups. CS and SW, on the other hand, were highly different from the control and LF, implying that the bacterial communities of CS and SW were very distinct from the control and LF. In addition, the UPGMA clustering results revealed that the four soil groups were primarily divided into two sections: control and LF, and CS and SW (data not shown). This finding also supports the notion that TPH contamination and the soil remediation process could have a significant impact on bacterial communities.

### 3.3. Varied Bacterial Distribution in Soil

**Figure 2:**
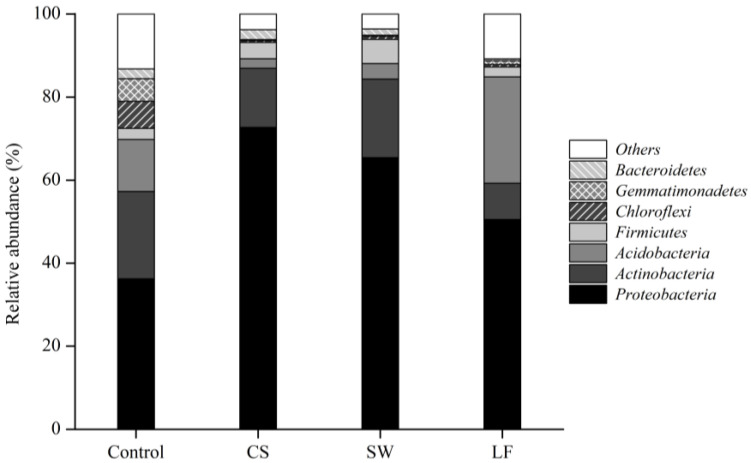
Relative abundance of soil bacteria in the four soil groups at the phylum level.

A total of 21 phyla, 53 classes, 119 orders, 242 families, 659 genera, and 1118 species were identified in all soil samples. Figure 2 represents the bacterial phyla with a relative abundance of 1% or more. The four soil groups consisted mainly of Proteobacteria, Actinobacteria, Acidobacteria, Firmicutes, Chloroflexi, Gemmatimonadetes, and Bacteroidetes. They accounted for more than 90% of the total bacteria present in the soil.

Despite the fact that the phylum composition of each group differed, Proteobacteria were identified in the largest proportion in all soils. The control group had the lowest rate at 36.22%, while CS had the highest rate at 72.72%. In addition, SW and LF decreased to 65.41% and 50.51%, respectively, after the soil remediation process. Proteobacteria are abundant in a variety of soil environments [41] and play an essential role in the carbon, sulfur, and nitrogen cycles in the soil [42]. Furthermore, many bacteria belonging to the phylum Proteobacteria degrade hydrocarbons in the soil [43]. The prevalence of Proteobacteria in TPH-contaminated soil has also been reported elsewhere [44,45]. Additionally, the largest proportion of Proteobacteria observed in CS is consistent with previous research demonstrating that TPH concentration and relative abundance of Proteobacteria increased proportionally [46,47]. Similar to the decrease in Proteobacteria in SW and LF, the degradation of TPH during the soil remediation process could be linked.

**Figure 3:**
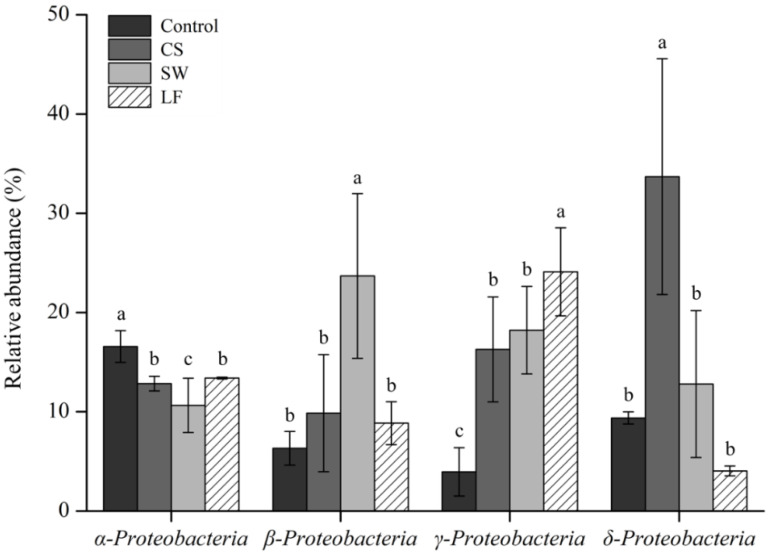
Differences in relative abundance of classes of Proteobacteria within the samples (Different letters in legend indicate significantly differences at *p* < 0.05).

In addition, among the four soil groupings, the distribution pattern of bacterial classes in the Proteobacteria phylum differed. Figure 3 shows the relative abundance of 0.1% or more among the seven classes of Proteobacteria (Alpha-, Beta-, Gamma-, Delta-, Epsilon-proteobacteria, Hydrogenophilalia, and Oligoflexia) observed in this study. Alphaproteobacteria were found in the range of 10.63–16.56% in all soil groups and were predominant in the control. This class was found most frequently in uncontaminated, ordinary soil, and Betaproteobacteria and Gammaproteobacteria were found in relatively low proportions [48,49]. Betaproteobacteria (6.32%), Gammaproteobacteria (3.94%), and Deltaproteobacteria (9.36%) were less abundant in the control group than Alphaproteobacteria (16.56%).

Deltaproteobacteria, on the other hand, were predominant in CS, and their prevalence increased significantly from 9.36% to 33.68% compared to the control. Additionally, Betaproteobacteria increased from 6.32% to 9.85%, while Gammaproteobacteria increased from 3.94% to 16.27%. These findings are consistent with previous research, which suggests that TPH-contaminated soil has a reduction in Alphaproteobacteria and an increase in Beta-, Gamma-, and Deltaproteobacteria [50,51]. The presence of several types of TPH-degrading bacteria belonging to Beta-, Gamma-, and Deltaproteobacteria [52], which play a significant role in the biodegradation of hydrocarbons in the soil [53], caused these bacterial composition modifications. Most TPH-degrading bacteria belong to the Gammaproteobacteria class, which results, in increased relative abundance at high TPH concentrations [54,55]. Many studies have labeled this phenomenon as a “gamma shift” [56,57,58]. Meanwhile, during the soil remediation process, the bacterial composition altered again. Deltaproteobacteria in SW and LF decreased to 12.78% and 4.04%, respectively, as compared to CS In addition, Betaproteobacteria increased to 23.68% in SW, whereas Gammaproteobacteria increased to 24.09% in LF.

Actinobacteria was the second most dominant phylum in the control (21.04%), CS (14.19%), and SW (18.90%). The relative abundance of Actinobacteria in LF, on the other hand, was 8.76%, which was lower than that in the other soil groups. Actinobacteria, Bacteroidetes, and Firmicutes contain TPH-degrading bacteria, such as Proteobacteria [59,60,61], and are commonly found in TPH-contaminated soil [62,63]. However, in contrast to previous studies, Actinobacteria was the most prevalent, accounting for 21.04% in the control and 14.19% in CS. In addition, despite differences in TPH concentration and remediation processes, the relative abundance of Bacteroidetes and Firmicutes did not differ in all soil groups (*p* < 0.05).

### 3.4. Effect of TPH-Degrading Bacteria in Contaminated and Remediated Soil

In this study, we tried to determine how TPH-degrading bacteria affect the bacterial community structure. Information on TPH-degrading bacteria was gathered from previous studies and their distribution by soil groups at the genus level was analyzed [64,65,66,67,68,69,70]. Table 3 shows the TPH-degrading bacteria, found in all soil samples, with a relative abundance greater than or equal to 0.01%. TPH-degrading bacteria mainly belonged to Proteobacteria, Actinobacteria, Firmicutes, and Bacteroidetes. These were present in the control and CS groups at 4.05% and 20.61%, respectively. Many soil bacteria act as TPH decomposers, using hydrocarbons in the soil as carbon and energy sources [71]. The release of TPH as a pollutant into the soil invariably increases the number of TPH-degrading bacteria [72]. Similarly, the relative abundance of TPH-degrading bacteria in the CS was more than three times higher than in the control in this study. In comparison to other TPH-degrading bacteria, Acinetobacter, Immundisolibacter, *Pseudomonas*, *Pseudoxanthomonas*, *Staphylococcus*, and *Thermomonas* had higher relative abundances in CS. These bacteria were frequently observed at relatively high TPH concentrations, which is consistent with previous results [73,74]. In addition, these bacteria were Proteobacteria, contributing to an increase in Gammaproteobacteria in CS compared to the control. Deltaproteobacteria however, was listed among the bacteria that degrade TPH. This suggests that the increase in Deltaproteobacteria in the CS was not caused by TPH-degrading bacteria. The percentage of TPH-degrading bacteria in SW was slightly higher than in the CS at 23.49%, which was slightly higher than that in the CS. When compared to CS, this group had a higher abundance of Acinetobacter, Immundisolibacter, *Pseudomonas*, and *Staphylococcus,* as well as a similar distribution pattern of TPH-degrading bacteria. Moreover, comparable to the similarity of beta diversity between CS and SW, these results suggest that the soil washing process did not notably affect the TPH-degrading bacteria or the overall bacterial community structure. The TPH-degrading bacteria in LF, on the other hand, decreased to 12.29%. The return of the bacterial population to the uncontaminated level (control group) following the landfarming process appears to be the cause of this decrease in relative abundance.

## 4. Conclusions

Changes in the soil environment, such as soil pollution or remediation processes can have a significant impact on soil biological properties. This study used metagenomic analysis to examine how bacterial diversity changed in TPH-contaminated soil after soil washing and landfarming remediation processes. The Illumina MiSeq platform was utilized to compare soil bacterial diversity in this study, which clearly demonstrated that bacterial species diversity varied depending on the soil remediation process.

In the presence of TPH in the soil, bacterial alpha diversity was reduced. LF removed less TPH than SW, but it vastly increased bacterial richness and diversity. Various distribution patterns for each soil group were identified as a result of this analysis of beta diversity and relative abundance at the phylum and class levels. The bacterial community of LF was determined to be similar to the control group. The many TPH-degrading bacteria found in SW, however, revealed that the soil washing process did not significantly affect the bacterial communities.

From the perspective of bacterial ecology, these results suggest that the landfarming process of TPH remediation is more advantageous than soil washing.

## Figures and Tables

**Table 1:** The result of chemical properties and total petroleum hydrocarbon (TPH) concentration in soil (mean ± SD ^†^).

Treatment	pH	Electric Conductivity (EC)	Soil Organic Matter (SOM)	Available Phosphorus (Av.P)	Cation Exchange Capacity (CEC)	Total Nitrogen (TN)	TPH
	1:5	dS m^−1^	%	mg kg^−1^	cmol_c_ kg^−1^	%	mg kg^−1^
Control	6.23 ± 0.58 ^b^	0.38 ± 0.06 ^a^	1.54 ± 0.04 ^a^	24.6 ± 11.2 ^a^	14.9 ± 4.00 ^b^	0.90 ± 0.36 ^a^	77 ± 17 ^b^
CS	6.22 ± 0.69 ^b^	0.29 ± 0.10 ^a^	0.90 ± 0.22 ^b^	21.7 ± 12.1 ^a^	8.91 ± 2.90 ^c^	0.09 ± 0.09 ^b^	2,690 ± 680 ^a^
SW	7.23 ± 0.11 ^a^	0.11 ± 0.02 ^b^	0.81 ± 0.02 ^b^	2.86 ± 1.57 ^b^	11.7 ± 2.40 ^bc^	0.13 ± 0.16 ^b^	111 ± 39 ^b^
LF	6.90 ± 0.06 ^ab^	0.28 ± 0.10 ^a^	1.06 ± 0.01 ^b^	18.7 ± 0.10 ^a^	37.4 ± 0.80 ^a^	0.93 ± 0.38 ^a^	249 ± 39 ^b^

^†^ All value is an average value of measurement, and different letters in rows indicate significantly differences at *p* < 0.05.

**Table 2:** The number of operational taxonomic units (OTUs), bacterial community richness and diversity estimators based on 16S rRNA gene (mean ± SD ^†^).

Sample Group	Reads	Good’s Coverage (%)	OTUs	Chao1	Shannon
Control	14,907 ± 2188 ^d^	97.99 ± 0.12	1265 ± 308 ^a^	1475 ± 340 ^a^	8.58 ± 0.62 ^a^
CS	35,782 ± 12,110 ^a^	99.95 ± 0.03	296 ± 13 ^c^	308 ± 24 ^c^	4.76 ± 1.30 ^c^
SW	29,979 ± 5923 ^b^	99.94 ± 0.08	305 ± 47 ^c^	316 ± 63 ^c^	5.94 ± 0.57 ^b^
LF	21,345 ± 3826 ^c^	98.99 ± 0.16	815 ± 111 ^b^	1000 ± 101 ^b^	7.06 ± 0.29 ^b^

^†^ All value is an average value of measurement, and different letters in rows indicate significantly differences at *p* < 0.05.

**Table 3:** Distribution of the well-known TPH-degrading bacterial genera in the soils ≥0.01%.

Phylum	Genus	Relative Abundance (%)			
Class		Control	CS	SW	LF
Actinobacteria					
Actinobacteria	*Corynebacterium*	0.01	0.85	1.20	-
	*Dietzia*	-	0.08	-	-
	*Kocuria*	0.06	0.10	-	-
	*Microbacterium*	0.02	0.31	0.03	0.01
	*Micrococcus*	-	0.33	0.05	-
	*Nocardioides*	0.90	1.41	0.82	0.24
	*Rhodococcus*	-	-	0.01	0.05
	*Streptomyces*	1.49	0.04	0.08	0.11
Bacteroidetes					
Flavobacteriia	*Chryseobacterium*	-	0.01	0.08	-
	*Flavobacterium*	0.03	0.04	0.09	-
Firmicutes					
Bacilli	*Bacillus*	0.26	0.29	0.32	0.93
	*Paenibacillus*	0.02	0.06	-	0.04
	*Staphylococcus*	0.03	1.37	3.39	-
	*Streptococcus*	-	0.52	0.92	-
Proteobacteria					
Alphaproteobacteria	*Azospirillum*	0.05	0.06	0.13	0.43
	*Methylobacterium*	0.17	0.19	0.13	0.01
	*Paracoccus*	-	0.77	0.03	-
	*Rhizobium*	0.24	0.09	0.52	0.02
Betaproteobacteria	*Acidovorax*	0.07	0.60	0.99	0.02
	*Burkholderia*	0.25	-	-	0.01
Gammaproteobacteria	*Acinetobacter*	0.03	2.92	5.54	-
	*Alkanindiges*	-	0.07	0.01	-
	*Immundisolibacter*	0.02	1.82	3.26	8.63
	*Pseudomonas*	0.15	1.72	3.38	0.05
	*Pseudoxanthomonas*	0.10	2.85	0.96	-
	*Rhodanobacter*	0.01	0.64	0.21	1.70
	*Stenotrophomonas*	0.04	0.03	0.10	-
	*Thermomonas*	0.10	3.44	1.25	0.04
Total		4.05	20.61	23.49	12.29

## Data Availability

The data presented in this study are available on request from the corresponding author.
